# Positioning of Melflufen in Heavily Pretreated RRMM Patients: Real‐World Evidence in a Rapidly Evolving Therapeutic Landscape

**DOI:** 10.1111/ejh.70156

**Published:** 2026-03-11

**Authors:** K. Mancuso, S. Masci, M. Talarico, A. Vitale, S. Barbato, V. Solli, M. Puppi, I. Rizzello, L. Pantani, P. Tacchetti, M. Iezza, R. Restuccia, I. Sacchetti, E. Manzato, S. Ghibellini, S. Armuzzi, B. Taurisano, M. Cavo, E. Zamagni

**Affiliations:** ^1^ IRCCS Azienda Ospedaliero‐Universitaria di Bologna Istituto di Ematologia “Seràgnoli” Bologna Italy; ^2^ Dipartimento di Scienze Mediche e Chirurgiche Università di Bologna Bologna Italy

**Keywords:** alkylating agents, melflufen, melphalan‐flufenamide, real‐world study, relapsed/refractory multiple myeloma

## Abstract

Modern therapies have clearly marked the history of multiple myeloma (MM), leading to undisputed advantages in terms of sustained responses and prolonged survival, while progressively improving patients' quality of life. Nonetheless, disease recurrence and resistance to available therapies underscore the importance of identifying additional treatment options, especially in hard‐to‐treat patients, or when access to cutting‐edge immunotherapies is limited. Melflufen (melphalan‐flufenamide) plus dexamethasone has been approved by the European Medicines Agency for triple‐class‐refractory MM patients after ≥ 3 prior therapies, but current data from real‐world settings are scarce. We herein report data from a single‐center experience of 17 relapsed/refractory MM patients treated with melflufen‐dexamethasone outside clinical trials between December 2021 and July 2025 in Bologna (Italy). The overall response rate was 41%. At a median follow‐up (mFU) of 8 months, mPFS was 3.7 months (95% CI 1.8–NR) in the overall population, being 9.0 months (95% CI 7.8–NR) in responders (mFU 10 months), while mOS has not been reached (95% CI 13.5–NR) either in the total population or in subgroups. Notably, 11/17 patients received subsequent therapies (seven receiving novel immunotherapeutic approaches), achieving deeper and more durable responses than those receiving conventional regimens. Grade ≥ 3 hematologic toxicities were common (35% anemia, 53% neutropenia, 53% thrombocytopenia), while grade ≥ 3 nonhematologic events were less frequent (mainly fatigue: 6%, and infections: 23.5%). No secondary primary malignancies were recorded. Collectively, our data confirmed the efficacy previously reported with melflufen–dexamethasone and its manageable safety profile, even in elderly patients likely more fragile and more heavily pretreated than those included in the trials. Overall, melflufen–dexamethasone may represent a treatment option, especially for patients refractory to novel immunotherapies or those who are not ideal candidates to receive such treatments while still preserving access to subsequent T‐cell redirecting therapies, thereby addressing a significant unmet need in this hard‐to‐treat patient population.

## 1 Introduction

Over the past two decades, advances in multiple myeloma (MM) therapy have improved response depth, survival, and quality of life. Nonetheless, despite the advances obtained by combining various classes of drugs—including proteasome inhibitors (PIs), immunomodulatory agents (IMiDs), and monoclonal antibodies (MoAbs)—the disease remains incurable, and the dismal prognosis of triple‐class refractory (TCR) patients underscores the urgent need for new treatment options [1, 2, 3, 4].

More recently, modern immunotherapeutic approaches, including antibody–drug conjugates (ADCs) and T‐cell redirecting therapies, led by chimeric antigen receptor T (CAR‐T) cells and bispecific antibodies (BsAbs), have brought a different perspective on the treatment paradigm for relapsed/refractory (RRMM) patients, but their use may be limited by logistical, socioeconomic, and toxicity‐related constraints [5, 6, 7, 8]. In such cases, expanding the treatment armamentarium with novel agents and alternative targets or mechanisms of action remains essential.

In this regard, the novel agent melphalan flufenamide (melflufen), a first‐in‐class lipophilic alkylating peptide–drug conjugate consisting of melphalan conjugated to the peptide *para*‐fluoro‐l‐phenylalanine, may have the potential to fill a gap among available anti‐MM therapies [9]. Unlike conventional alkylating agents used to date in MM, melflufen has shown greater permeability through the cell membrane, resulting in higher—and faster—intracellular concentration of melphalan and increased cytotoxic activity. Indeed, its lipophilic nature ensures rapid absorption into cells, where it is cleaved by aminopeptidases to release the hydrophilic alkylator payloads, thereby causing irreversible DNA damage and cell death [10].

Following preclinical [11] and early‐phase studies to determine dose (phase I/II O‐12‐M1 study) [12] and its pharmacokinetic profile, also including patients with reduced renal function (phase II OP‐107 trial) [13], the efficacy of melflufen in association with dexamethasone in MM has been documented in the pivotal phase II HORIZON (NCT02963493), leading to the accelerated approval (February 26, 2021) in TCR RRMM patients previously treated with at least 4 lines of therapy by the US Food and Drug Administration (FDA) [14]. Briefly, 157 RRMM patients refractory to pomalidomide and/or a CD38‐targeting MoAb after at least 2 prior PI‐ and IMiD‐containing therapies were enrolled. The overall response rate (ORR) was 29% in the all‐treated population who had median (m) progression‐free survival (PFS) and overall survival (OS) values of 4.2 and 12 months, respectively [15]. Efficacy of this combination was also confirmed by subgroup analyses of patients with high‐risk cytogenetics or extramedullary disease (EMD) [16, 17].

Subsequently, 495 lenalidomide‐refractory patients with 2–4 prior therapies including lenalidomide and a PI were randomized in the phase III OCEAN (NCT03151811) trial to receive either melflufen–dexamethasone or pomalidomide–dexamethasone (Pd), resulting in 33% vs. 27% ORR (*p* = 0.16) and 6.8 vs. 4.9 months mPFS (HR = 0.79, *p* = 0.032) [18]. However, due to the inferior mOS in the intention‐to‐treat population (20.2 vs. 24 months, HR = 1.1, *p* = 0.24), the combination was withdrawn in the US (September, 2022) [19], though further post hoc analyses showed a clinical benefit in the melflufen‐dexamethasone arm when excluding patients who had previously undergone ASCT or who had progressed within 3 years from transplantation. Indeed, updated analyses showed 23.6 vs. 19.8 months mOS (HR = 0.83, *p* = 0.225) in patients with no prior ASCT or progressing after 36 months (melflufen‐dexamethasone vs. Pd), mPFS of 9.3 vs. 4.6 months (HR = 0.58, *p* = 0.0001), and 42% vs. 26% ORR. In line with these data, the combination melflufen‐dexamethasone is approved by the European Medicines Agency (EMA) for the treatment of TCR patients who have received at least three previous treatment lines and progressed on/after last therapy; for patients with prior ASCT, the time‐to‐progression (TTP) should be superior to 3 years from transplantation [20].

In this scenario, gathering information from real‐world evidence to reinforce data on the clinical efficacy of this combination and assess its safety in real‐life settings is essential. To this aim, we herein present data from a single‐center experience of RRMM patients treated with melflufen plus dexamethasone outside clinical trials and discuss these data in light of the current treatment paradigm in MM.

## 2 Materials and Methods

### 2.1 Study Design

This retrospective analysis included patients treated in real life with melflufen‐dexamethasone at the UOC Ematologia, IRCCS Azienda Ospedaliero‐Universitaria di Bologna, Policlinico di S.Orsola (Bologna, Italy) (IRCCS AOUBO) between December 2021 and July 2025.

Eligible patients were aged ≥ 18 years, had a confirmed diagnosis of MM according to International Myeloma Working Group (IMWG) criteria [21], presented with relapsed/refractory disease after exposure to the three major drug classes, and received melflufen‐dexamethasone in a real‐world, nonclinical trial setting. Treatment included intravenous melflufen 40 mg on Day 1 of each 28‐day cycle and oral dexamethasone 40 mg (20 mg if ≥ 75 years) on Days 1, 8, 15, and 22 [22].

The study was approved by Comitato Etico Area Vasta Emilia Romagna (CE‐AVEC) and was conducted in accordance with the International Conference on Harmonization Guidelines on Good Clinical Practice and the principles of the Declaration of Helsinki. All participants signed an informed consent form prior to their inclusion in the study.

Data on patients' baseline characteristics and prior treatments, disease status, melflufen/dexamethasone dose and schedule, responses, hematologic and nonhematologic toxicities, subsequent and supportive therapies were collected and analyzed with descriptive statistics. Responses were assessed using the IMWG uniform response criteria [23].

### 2.2 Statistical Analysis

All consecutive patients meeting all the eligibility criteria were enrolled in the study and registered in an e‐CRF web‐based database. Given the low number of samples, descriptive statistics were presented for all the study variables; categorical variables were summarized by using absolute and relative frequency distribution, expressed in percentage; continuous variables were summarized by mean and standard deviation or median, min, max, and interquartile range (IQR), as appropriate, to provide a robust description of data, preserving their nonparametric nature.

The treatment regimens used, in terms of drug dose, duration of treatment, interruptions, dose reductions, and concomitant therapies, were presented by means of descriptive statistics as well. Survival curves were estimated according to Kaplan Meier's method, calculating both the median TTP and time to survival. The hazard ratio (HR) risk measure indicator was obtained using a semiparametric Cox regression model. The significance level for this analysis was set at 0.10 (CI 90%). All analyses were performed using R‐Studio version 4.3.3.

Treatment‐related AEs were collected and reported according to the Common Terminology Criteria for AEs (CTCAE), Version 5.0.

## 3 Results

### 3.1 Patient Characteristics and Pre‐Treatment Status

Patient baseline characteristics and prior treatment status are summarized in Table 1. Seventeen patients with RRMM were included, with a median age of 71 years (range 59–84); notably, 7 patients (41%) were ≥ 75 years old. Regarding major high‐risk features, one patient (6%) presented with EMD, one (6%) had circulating plasma cells exceeding 5%, and 7 (41%) harbored high‐risk cytogenetics (including del17p, *t*(4;14), *t*(14;16), or 1q gain/amplification).

**TABLE 1:** Baseline characteristics, prior therapies, and refractoriness status.

Characteristic	*N* = 17
Female, *n* (%)	8 (47)
Median age, years (range)	71 (59–84)
65–74 years, *n* (%)	6 (35)
≥ 75 years, *n* (%)	7 (41)
ECOG 0, *n* (%)	14 (82)
ECOG 1, *n* (%)	3 (18)
Cytogenetics abnormalities, *n* (%)	
High‐risk^a^	7 (41)
Standard risk	3 (18)
NA	7 (41)
EMD, *n* (%)	1 (6)
Median time from diagnosis to treatment, years (range)	6 (1–23)
Median n. of prior treatments (range)	4 (2–11)^b^
Prior ASCT, *n* (%)	5 (29)
Double‐class refractory, *n* (%)	17 (100)
Triple‐class exposed, *n* (%)	17 (100)
Triple‐class refractory, *n* (%)	15 (88)
Penta‐class refractory, *n* (%)	7 (41)
Refractoriness to specific drugs, *n* (%)	
Lenalidomide	14 (82)
Pomalidomide	15 (88)
Bortezomib	10 (59)
Carfilzomib	12 (71)
Anti‐CD38	17 (100)
Exposure to anti‐BCMA therapies, *n* (%)	5 (29)
Refractoriness to anti‐BCMA therapies	5 (29)
Belantamab mafodotin	3 (18)
Belantamab mafodotin and teclistamab	1 (6)
Ide‐cel and teclistamab	1 (6)
Exposure to anti‐GPRC5D BsAb, *n* (%)	1 (6)
Refractoriness to anti‐GPRC5D, *n* (%)	1 (6)
Median creatinine clearance, mL/min (range)	69 (11–97)
≥ 45 mL/min < 60 mL/min, *n* (%)	3 (18)
mL/min	3 (18)

Abbreviations: ASCT, autologous stem cell transplantation; BsAb, bispecific antibody; EMD, extramedullary disease; NA, not available.

^a^ High‐risk cytogenetics was defined as the presence of one of the followings: del17p, *t*(4;14), *t*(14;16), or 1q gain/amplification.

^b^ The lower limit of two prior lines reflects one patient presenting with aggressive, triple‐refractory disease, for whom no alternative clinically feasible treatment option was available at the time of relapse.

The median time from diagnosis was 6 years (range 1–23) and the median number of prior treatment lines was four (range 2–11). All patients were triple‐class exposed, while 15 (88%) were TCR and seven (41%) were penta‐drug refractory. Among the 17 patients, 4 (24%) had previously been exposed to melphalan: notably, none of these patients had received melphalan in the line of therapy immediately preceding melflufen, as melphalan exposure occurred in earlier treatment lines, often several lines before melflufen initiation (e.g., as part of frontline therapies). Importantly, five patients (29%) had been previously exposed and were refractory to anti‐BCMA immunotherapies, and one (6%) among these was also refractory to an anti‐GPRC5D BsAb. Two patients (12%) were refractory to selinexor. Five patients (29%) had undergone ≥ 1 prior ASCT, with a medium interval of 33 months from transplant to the initiation of melflufen. Of note, three of these patients had progressed within 36 months from transplantation: in the absence of valid alternative effective treatment options, it was decided to initiate treatment with melflufen in these patients.

Notably, a subset of patients presented with reduced renal function at baseline: the median creatinine clearance was 69 mL/min/1.73 m^2^ (range 11–37), with six patients having a clearance < 60 mL/min, including three with values < 45 mL/min, which is below the threshold required in the pivotal registration trials. Despite the advanced disease stage, patients had preserved bone marrow reserve at treatment initiation, as indicated by median baseline counts of neutrophils 3040/mm^3^ (range 1470–6260), platelets 180 000/mm^3^ (range 75 000–313 000), and hemoglobin 11.6 g/dL (range 7.7–14.9).

### 3.2 Therapy Administration and Outcomes

Melflufen was administered to all patients via central‐venous‐catheter, placed before therapy initiation. Eight patients (47%) received the standard starting dose of 40 mg, six (35%) received a reduced dose of 30 mg, in accordance with the label recommendations for patients with body weight ≤ 60 kg or eGFR 30–45 mL/min/1.73 m^2^, and one patient (6%) received 10 mg due to severe renal impairment (eGFR < 30 mL/min/1.73 m^2^), though data to support a dose recommendation in these latter cases are limited [22]. Only one (6%) patient received the full 40 mg starting steroid dose; the others received 20 mg or less owing to age, comorbidities, or prior steroid intolerance. The median number of treatment cycles received was three (range 1–9). At the time of analysis, three patients were still on treatment. Reasons for discontinuation included disease progression (*n* = 11.65%), AEs (*n* = 2, 12%, including one fatal case due to septic shock), and planned CAR‐T therapy (*n* = 1.6%). Overall, response was assessable in 94% of patients (*n* = 16), while one died from septic shock prior to disease reassessment. The ORR was 41% (*n* = 7), with median time to best response of 2 months (range 1–12). Specifically, two patients (12%) achieved complete remission (CR) and five (29%) partial response (PR). In addition, three patients (18%) obtained minimal response, two (12%) had stable disease, and four (23%) experienced progressive disease.

**FIGURE 1:**
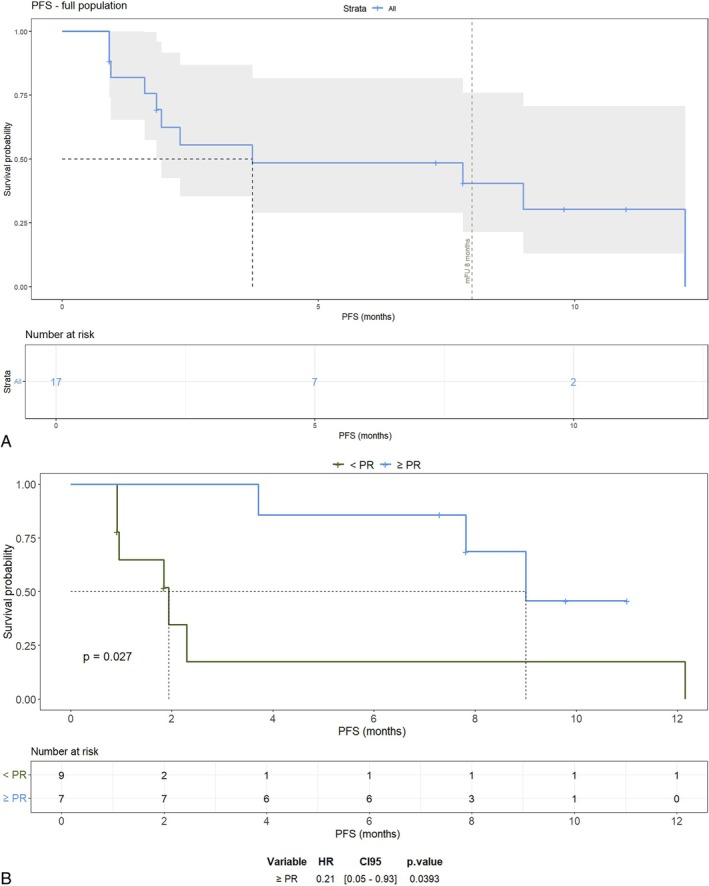
Progression‐free survival in the general population (A) and among responders (≥ PR) (B).

At a median follow‐up (mFU) of 8 months, mPFS in the overall population was 3.7 months (95% CI 1.8–NR). Responders (≥ PR) experienced significantly improved outcomes, with mPFS of 9.0 months (95% CI 7.8–NR, mFU 10 months) compared with 1.8 months (95% CI 0.9–NR mFU 8 months) in patients achieving less than PR (*p* = 0.027; HR = 0.21, *p* = 0.039) (Figure 1). Median duration of response (DOR) in the overall population was 2.57 months (95% CI 0–9.93), 3.43 months among responders (95% CI 1.87–9.93). Median OS has not been reached (95% CI 13.5–NR) either in the total population or in the subgroups (Figure S1), being 76.5% at mFU.

Notably, no statistically significant differences were observed in achieving responses ≥ PR versus < PR according to the presence of high‐risk cytogenetics, prior ASCT, prior exposure to melphalan, TTP after ASCT < 36 months, triple‐ or penta‐refractory status, or refractoriness to anti‐BCMA therapies and GPRC5D‐targeted treatments (Table S1).

Due to the limited sample size, no formal statistical inference or further correlative (univariate) analyses were performed, as the statistical power would not have been sufficient to allow for reliable interpretation.

### 3.3 Safety Profile

The main hematologic and nonhematologic toxicities are listed in Table 2.

**TABLE 2:** Hematologic and nonhematologic toxicities and need for supportive care.

	All grades	Grade 1	Grade 2	Grade 3	Grade 4	Grade 5
Hematologic toxicities						
Anemia, *n* (%)	9 (53)	1 (6)	2 (12)	6 (35)	—	—
Neutropenia, *n* (%)	10 (59)	1 (6)	—	5 (29)	4 (24)	—
Thrombocytopenia, *n* (%)	11 (65)	1 (6)	1 (6)	3 (18)	6 (35)	—
Nonhematologic toxicities						
Fatigue, *n* (%)	12 (71)	7 (41)	4 (24)	1 (6)	—	—
Nausea, *n* (%)	4 (24)	4 (24)	—	—	—	—
Vomiting, *n* (%)	1 (6)	1 (6)	—	—	—	—
Diarrhea, *n* (%)	—	—	—	—	—	—
Infections, *n* (%)	7 (41)	1 (6)	2 (12)	3 (18)	—	1 (6)
SPM, *n* (%)	—	—	—	—	—	—

	Yes	No
Supportive care		
RBC transfusions, *n* (%)	6 (35)	11 (65)
PLT transfusions, *n* (%)	4 (24)	13 (77)
G‐CSF support, *n* (%)	10 (59)	7 (41)

Abbreviations: G‐CSF, granulocyte‐colony stimulating factor; PLT, platelets; RBC, red blood cells.

Hematologic toxicities of grade ≥ 3 were common, with anemia occurring in six (35%) patients, neutropenia in nine (53%), and thrombocytopenia in nine (53%). Nonhematologic grade ≥ 3 events were less frequent and consisted mainly of fatigue in one (6%) patient and infections in four (23.5%), leading to treatment discontinuation in two (12%) patients: one due to septic shock, which was fatal, and the other due to severe pneumonia and rectal bleeding.

Supportive care (Table 2) was frequently required, with six (35%) patients receiving red blood cell transfusions, four (24%) platelet transfusions, and 10 (59%) granulocyte colony‐stimulating factor (G‐CSF) support. Due to hematologic toxicity, treatment administration was delayed at least once in seven (41%) patients. No secondary primary malignancies were recorded during follow‐up. Overall, the safety profile was consistent with previous reports of melflufen and reflects the frailty of this real‐world cohort.

### 3.4 Subsequent Therapies

A detailed summary of subsequent therapies, best response, and duration for each regimen is provided in Table S2. Among the 11 (65%) patients who received subsequent treatment after melflufen discontinuation, the median time from the last infusion to the initiation of the next therapy was 2.5 months (range 1–9). Notably, at least seven (64%) of these patients were treated with novel immunotherapies in later lines. Specifically, six (55%) received immunotherapy immediately after melflufen: two with belantamab mafodotin, two with teclistamab, one with elranatamab, and one with CAR‐T therapy (in this latter case, melflufen was used as a bridging treatment). The remaining patients received nonimmunotherapy regimens, including three with selinexor–bortezomib–dexamethasone (SVd), one with mezigdomide–cyclophosphamide–dexamethasone, and one with pomalidomide–dexamethasone (Pd).

Regarding treatment outcomes, all patients exposed to post‐melflufen immunotherapies achieved at least a PR, all but one reaching very good partial response (VGPR) or better; of note, many of these responses were still ongoing at the last patient follow‐up (Table S2). By contrast, standard regimens such as SVd or Pd yielded suboptimal results, with three patients experiencing early progressive disease (two with SVd, one with Pd) and one achieving stable disease only (SVd). Notably, the mezigdomide‐based regimen induced a VGPR in one patient (still on treatment at the last follow‐up). At a median follow‐up of 12 months, mPFS for the overall cohort of 11 patients treated after melflufen was 3 months (95% CI 1.8–NR), while mOS was not reached (95% CI 13.5–NR). Of note, among those who received an immunotherapy‐based regimen in subsequent lines, mPFS at a median follow‐up of 14 months was 8 months (95% CI 1.8–NA). Importantly, most patients treated with novel immunotherapies were still in response at data cut‐off (July 31, 2025), whereas those treated with standard regimens showed only transient or no benefit.

## 4 Discussion and Conclusions

The therapeutic landscape of RRMM has evolved significantly in recent years, with novel immunotherapies such as CAR‐T cells and BsAbs now prioritized in treatment algorithms [23, 24]. However, while an increasing number of patients are able to access these advanced therapies, their use in clinical practice may still be influenced by factors such as eligibility criteria, logistical challenges, center accreditation requirements (for CAR‐Ts) and costs [25, 26, 27]. Indeed, T‐cell redirecting therapies require multidisciplinary expertise and management, and are associated with significant toxicities, making careful risk–benefit assessment essential and limiting their suitability in frail, elderly, or comorbid patients. Consequently, an unmet therapeutic need is still present in this population. In addition, although novel immunotherapies are rapidly becoming the standard of care for TCR patients, there remains a significant unmet need before, between, and after treatment with BCMA‐ and GPRC5D‐targeted therapies. The optimal sequencing of these agents is still under investigation, and effective bridging or interval treatments are crucial to maintain disease control, prevent clinical deterioration, and allow patients to access subsequent lines of immunotherapy, or bridge them to the subsequent availability of drugs under approval.

In this context, melflufen may still represent a relevant treatment option for heavily pretreated patients, and the latest EHA‐EMN guidelines recognize its use in TCR patients who are either ASCT‐naïve or those who relapse ≥ 3 years post‐ASCT, as supported by high‐grade evidence [24]. Indeed, melflufen remains one of the few available treatment options for patients who are also refractory to CAR‐T cell therapies or ADCs, particularly when no other therapies are feasible [24]. In these more challenging clinical scenarios of advanced disease, the inclusion of melflufen among available options highlights the importance of maintaining flexible therapeutic approaches in real‐world clinical practice, where treatment strategies must frequently adapt to multifaceted and individualized patient needs. Moreover, the long‐term responses reported with melflufen‐dexamethasone in some cases, although exceptional, underscore the importance of additional available options in certain circumstances, filling an important gap and offering a glimmer of hope when no other alternatives can be found [28].

However, due to the lack of FDA approval, real‐world data on melflufen remain limited, underscoring the need for independent evidence outside clinical trials. Furthermore, real‐world studies capture patient populations underrepresented in clinical trials, including elderly, frail, or comorbid patients, and reflect contemporary RRMM cohorts that differ from earlier trial populations in terms of prior treatments and refractoriness. Against this backdrop, our study aimed to evaluate the effectiveness and tolerability of melflufen in a real‐life setting of heavily pre‐treated RRMM, providing insights into its role in the context of currently evolving therapeutic algorithms.

As an instance, our patient population presented distinctive features compared to those included in the phase II HORIZON and phase III OCEAN trials [14, 17]. Among the most relevant aspects, patients in our cohort were generally older, with a median age of 72 years (65 and 68 years in the HORIZON and OCEAN, respectively) and about half of them exceeding 75 years (13% OCEAN, 16% HORIZON). Moreover, our cohort of patients was more heavily pretreated, not so much in terms of the number of previous lines (four median prior lines, compared to the five and three in HORIZON and OCEAN, respectively), but rather in terms of resistance to multiple drug classes. Specifically, all patients included in our analysis were triple‐class exposed and 88% TCR (76% in the HORIZON and 16% in the OCEAN), being all refractory to anti‐CD38 MoAbs (80% in HORIZON, 20% in OCEAN), and nearly one‐third already exposed and refractory to novel immunotherapies, a population not represented in either trial, but increasingly common in real‐world practice. Overall, these aspects highlight the higher therapeutic pressure of our cohort with respect to previous reported data and emphasize the unmet need of current MM patient populations, in the present treatment scenario. Taken together, this advanced stage of therapeutic refractoriness likely reflects the evolution of treatment paradigms in recent years—especially the widespread adoption of anti‐CD38 MoAbs as core components of modern regimens, the earlier introduction of pomalidomide, and the increasing use of novel immunotherapies—and may suggest a population characterized by more aggressive and harder‐to‐treat disease. Nevertheless, despite the advanced clinical profile of our patients, their biological features, and the incidence of EMD were broadly in line with those reported in the registration trials. Specifically, high‐risk cytogenetic abnormalities were present in 47% of our cohort, a proportion slightly higher than that reported in HORIZON (38%) and OCEAN (34%). Only one patient (7%) presented with EMD at treatment initiation, compared with 35% in HORIZON and 13% in OCEAN. These findings suggest that the high degree of refractoriness observed in our population is more likely related to prior treatment intensity and cumulative drug exposure rather than to markedly adverse disease biology.

Overall, melflufen confirmed previous data in terms of efficacy, with 41% ORR (29% in HORIZON and 33% in OCEAN), despite the older age and multi‐class refractoriness of our cohort. Also, our study showed comparable mPFS (3.7 months) with the HORIZON trial (4.2 months), while mOS was not reached (HORIZON: 11.6 months). By contrast, the outcomes were slightly inferior to those observed in the OCEAN trial (mPFS 6.8 months; mOS 19.8 months), likely attributable to the markedly less pretreated and less refractory population enrolled in the trial compared with our cohort. Notably, response was not influenced by the presence of high‐risk cytogenetics, prior ASCT, prior exposure to melphalan, or refractoriness to previous drugs, including anti‐BCMA‐ or GPRC5D‐targeting treatments, thereby supporting a role for melflufen in real‐world later‐lines contexts and within modern therapeutic sequencing. Regarding safety, melflufen demonstrated an overall manageable safety profile, with hematologic AEs representing the most frequent and clinically relevant complications, in line with previous data, underscoring the reproducibility of this safety pattern across different patient populations. Importantly, no cases of secondary primary malignancies were observed during follow‐up.

Notably, 11 patients in our cohort received at least one subsequent line of therapy after melflufen, more than half with immunotherapy‐based regimens, achieving deeper and more durable responses than those receiving conventional therapies. Consistently, though median PFS in these patients was 3 months overall, it was approximately 8 months among those receiving subsequent immunotherapy, suggesting that exposure to melflufen does not preclude access to further immunotherapy‐based strategies. Rather than competing with emerging T‐cell‐engaging therapies, melflufen may provide meaningful disease control and serve, in selected cases, as a potential bridging option to such treatments—particularly when immediate access to CAR‐T cells or BsAbs is limited by nonavailability or temporary ineligibility—or represent an intermediate option, allowing immunologic wash‐out between sequential T‐cell–engaging therapies, thereby supporting its integration into a rational sequencing strategy. Prospective validation is warranted, especially considering the emerging concerns regarding potential negative impact of alkylating agents on lymphocyte apheresis prior to CAR‐T cell manufacturing, and the lack of evidence on the role of melflufen in the context of the widely debated issue of T‐cell exhaustion [23, 25, 27].

In addition to these considerations, and despite the more modest results when compared with T‐cell redirecting therapies, the use of melflufen requires fewer logistical resources than more advanced regimens, both in terms of patient management and complications handling. As a ready‐to‐use formulation, melflufen may indeed offer practical advantages in terms of dosing, schedule, and administration, thereby facilitating adherence to treatment with an accessible/simplified schedule of short monthly infusions from the start of therapy, making this combination suitable for frail or elderly patients, for whom access to immunotherapies is limited. In addition, though all patients in our study received melflufen via a central venous catheter, data from the PORT study (NCT04412707) demonstrated that the drug can also be administered through peripheral veins, thereby improving patient convenience and reducing the need for invasive procedures [29].

To date, however, very little data is available on its use outside clinical trials. To the best of our knowledge, there is currently only one study in the literature, reporting data on 12 RRMM patients (11 evaluable) only, none of whom had previously been exposed to anti‐BCMA or anti‐GPRC5D therapies, unlike our cohort. In addition to this, few trials have addressed the efficacy of melflufen combined with other agents (e.g., daratumumab or bortezomib) so far, as in the phase 1/2a ANCHOR trial (NCT03481556), or in the phase 3 LIGHTHOUSE (NCT04649060), this latter prematurely ended due to a partial clinical hold by FDA for all melflufen‐containing studies.

Certainly, our analysis harbors some limitations, including the retrospective design of the study, which limits the possibility of adequate patient selection, and the small number of patients included, limiting the statistical power and generalizability of our findings, and further subgroups analyses. Additionally, the still limited follow‐up prevented a robust assessment of long‐term outcomes, while data on subsequent therapies reflect the high variability of treatment regimens in advanced disease, largely dictated by the need to identify therapies with new mechanisms of action, balanced by their actual availability in this rapidly evolving therapeutic landscape. Not least, given the lack of alternatives, our cohort included three patients whose duration of response to ASCT was shorter than currently recommended thresholds for treatment eligibility.

In conclusion, our real‐world study confirmed the efficacy previously reported with melflufen–dexamethasone, with comparable results to other available therapies (i.e., selinexor‐based), and a manageable safety profile, that is retained in elderly patients, likely more fragile and more heavily pretreated than those included in the trials. Furthermore, the inclusion of patients exposed—or refractory—to novel immunotherapies has provided insights into a population that had never been considered before. Meanwhile, the use of melflufen in less heavily pretreated populations or in combination strategies with other drugs should be further investigated, and research in this sense is ongoing. Importantly, despite its modest results compared to more advanced therapies, this combination may still represent a treatment option, not only for patients refractory to novel immunotherapies, but also for those who are not ideal candidates to receive such treatments, while still preserving access to subsequent T‐cell redirecting therapies, thereby addressing a significant unmet need in this hard‐to‐treat patient population.

## Author Contributions

K.M. designed the research study, collected the data, analyzed the data, and wrote the paper. S.M. collected the data, analyzed the data, and wrote the paper. A.V. and V.S. performed the statistical analysis, and critically revised the paper. S.B. discussed the results, wrote the paper, and offered editorial support. M.T., M.P., I.R., L.P., P.T., M.I., R.R., I.S., and S.G. collected the data, discussed the results, and critically revised the paper. M.C. and E.Z. designed the research study, analyzed the data, discussed the results, and critically revised the paper. All authors contributed to the acquisition, analysis, or interpretation of data. All authors reviewed the paper and approved the final version to be published.

## Funding

The authors have nothing to report.

## Ethics Statement

The study was approved by CE AVEC and was conducted in accordance with the International Conference on Harmonization Guidelines on Good Clinical Practice and the principles of the Declaration of Helsinki. All participants signed an informed consent form prior to their inclusion in the study.

## Conflicts of Interest

K. Mancuso has received honoraria from Celgene, Takeda, Amgen, Sanofi and Janssen. I. Rizzello has received honoraria from Amgen, GlaxoSmithKline and Sanofi and advisory role for GlaxoSmithKline and Menarini Stemline. L. Pantani has received honoraria from GlaxoSmithKline, Pfizer and Sanofi. P. Tacchetti has received honoraria from Amgen, Bristol‐Myers Squibb/Celgene, Janssen, Takeda, AbbVie, Sanofi, GlaxoSmithKline and Pfizer. M. Cavo has received honoraria and has served in a consulting/advisory role for Amgen, AbbVie, Bristol‐Myers Squibb, Celgene, GlaxoSmithKline, Janssen, Menarini Stemline, Sanofi, and Karyopharm Therapeutics. E. Zamagni has received honoraria and has served in advisory role for Janssen, Bristol‐Myers Squibb, Sanofi, Amgen, GlaxoSmithKline, Pfizer, Oncopeptides, Menarini‐Stemline. S. Masci, M. Talarico, A. Vitale, S. Barbato, V. Solli, M. Puppi, M. Iezza, R. Restuccia, I. Sacchetti, E. Manzato, and S. Ghibellini declare no potential conflicts of interest.

## Supporting information


**Data S1:** ejh70156‐sup‐0001‐Supinfo.docx.

## Data Availability

The data supporting the findings of this study are available via the Zenodo platform (10.5281/zenodo.17453872).
