# Supplementary material for: Positioning of Melflufen in Heavily Pretreated RRMM Patients: Real‐World Evidence in a Rapidly Evolving Therapeutic Landscape
Source: Eur J Haematol. 2026 Mar 11;116(6):928–36. doi: 10.1111/ejh.70156 (PMC13141662; doi:10.1111/ejh.70156)
Supplement: Supplementary file 1 — Data S1: ejh70156‐sup‐0001‐Supinfo.docx. [file EJH-116-928-s001.docx]

**Supplementary**

**Figure S1 Overall survival in the general population (A) and by response category (B)**

**A**
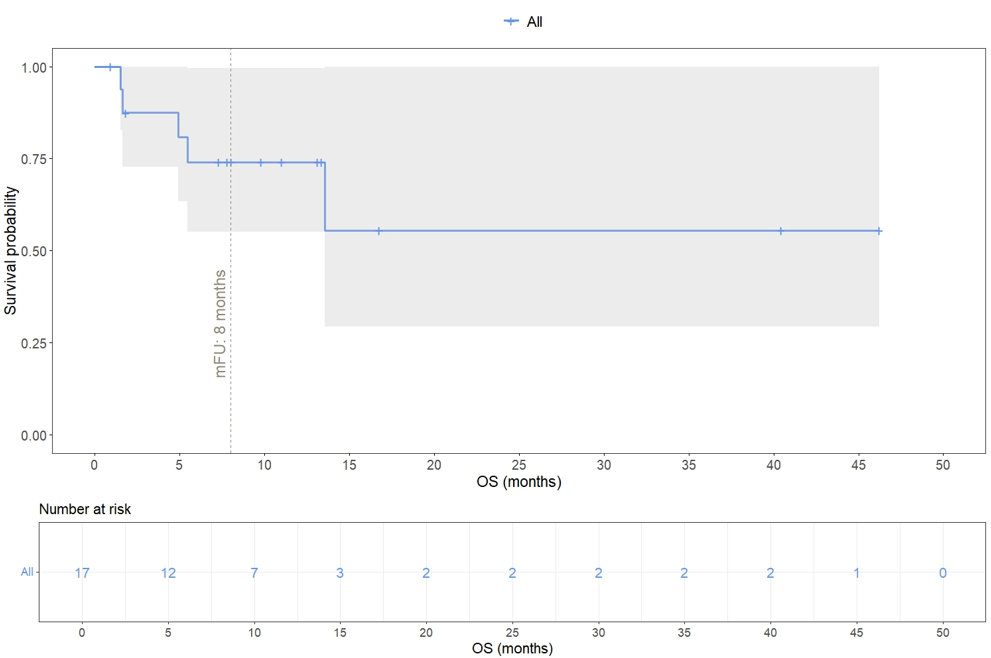


**B**
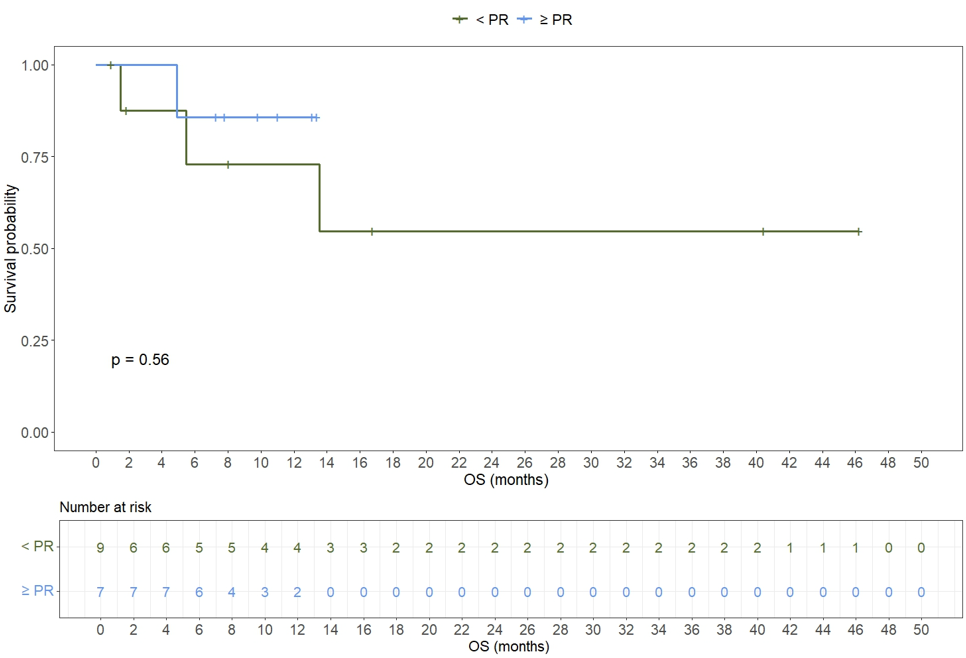


**Table S1 Response to therapy (≥PR or <PR) in specific patient subgroups**

| **Characteristic (N=16)** | **< PR (N = 9)** | **≥ PR (N = 7)** | **p-value** |
| --- | --- | --- | --- |
| High-risk cytogenetics  yes, n (%)  no, n (%)  NA, n (%) | 5 (71) 2 (29) 2 | 1 (50) 1 (50) 5 | >0.9 |
| Baseline EMD/PCL  yes, n (%)  no, n (%) | 1 (11)  8 (89) | 0 (0)  7 (100) | >0.9 |
| Previous ASCT  yes, n (%)  no, n (%) | 2 (22)  7 (78) | 2 (29)  5 (71) | >0.9 |
| TTP from ASCT ≤36 months  yes, n (%)  no, n (%)  NA, n (%) | 1 (50)  1 (50)  7 | 1 (50) 1 (50)  5 | >0.9 |
| Triple-refractoriness  yes, n (%)  no, n (%) | 9 (100)  0 (0) | 5 (71)  2 (29) | 0.2 |
| Penta-refractoriness  yes, n (%)  no, n (%) | 5 (56)  4 (44) | 1 (14)  6 (86) | 0.15 |
| Anti-BCMA exposure  yes, n (%)  no, n (%) | 4 (44)  5 (55) | 1 (14)  6 (86) | 0.6 |
| Anti-GPRC5D exposure  yes, n (%)  no, n (%) | 1 (11)  8 (89) | 0 (0)  7 (100) | >0.9 |

Statistical test: Fisher’s exact test

Abbreviations: ASCT= autologous stem-cells transplantation; BCMA= B-cell maturation antigen; EMD= extra-medullary disease; GPRC5D=; N= number; NA= not available; PCL= plasma-cell leukemia; PR= partial response; TTP= time to progression.

**Table S2 Subsequent therapies, best response achieved and duration of treatment with each regimen**

| **Patient**  (N=11) | **Next Therapy 1**  (Duration/ Best response) | **Next Therapy 2**  (Duration/ Best Response) | **Next Therapy 3** (Duration/Best Response) | **Status at Last Contact** |
| --- | --- | --- | --- | --- |
| 1 | Elranatamab  (3 cycles, CR) | - | - | CR, on treatment |
| 2 | Belantamab mafodotin  (3 cycles, CR) | - | - | CR, on treatment |
| 3 | SVd  (1 cycle, PD) | - | - | Death from PD |
| 4 | Mezi-Cyclo-Dex  (5 cycles, VGPR) | - | - | VGPR, on treatment |
| 5 | Belantamab mafodotin  (31 cycles, VGPR) | SVd  (3 cycles, SD) | Talquetamab  (2 cycles-ongoing, PR) | PR, on treatment |
| 6 | Teclistamab  (11 cycles, CR) | - | - | CR, off treatment  (teclistamab stopped due to infections) |
| 7 | Poma-Dex  (1 cycle, PD) | - | - | Death from PD |
| 8 | SVd  (3 cycles, SD) | Elranatamab  (8 cycles, CR) | Talquetamab  (2 cycles -ongoing, CR) | CR, on treatment |
| 9 | Ide-Cel  (5 months post-CAR-T CR) | - | - | CR, off treatment |
| 10 | Teclistamab  (3 cycles, VGPR) | - | - | Death from sepsis (VGPR) |
| 11 | SVd  (1 cycle, PD) | - | - | PD, off treatment |

Abbreviations: CR= complete response; Mezi-Cyclo-Dex= mezigdomide + cyclophosphamide + dexamethasone; N= number; PD= progressive disease; Poma-dex= pomalidomide + dexamethasone; SVd= selinexor + bortezomib + dexamethasone; VGPR= very good partial response.
